# Iron Deposition Leads to Hyperphosphorylation of Tau and Disruption of Insulin Signaling

**DOI:** 10.3389/fneur.2019.00607

**Published:** 2019-06-19

**Authors:** Wenbin Wan, Lan Cao, Bill Kalionis, Padma Murthi, Shijin Xia, Yangtai Guan

**Affiliations:** ^1^Department of Neurology, Renji Hospital, Shanghai Jiao Tong University School of Medicine, Shanghai, China; ^2^State Key Laboratory of Medical Neurobiology, Institutes of Brain Science, Shanghai Medical College, Fudan University, Shanghai, China; ^3^Department of Maternal-Fetal Medicine, Pregnancy Research Centre, University of Melbourne, Parkville, VIC, Australia; ^4^Department of Obstetrics and Gynecology, Royal Women's Hospital, Parkville, VIC, Australia; ^5^Department of Obstetrics and Gynecology, University of Melbourne, Parkville, VIC, Australia; ^6^Shanghai Institute of Geriatrics, Huadong Hospital, Fudan University, Shanghai, China

**Keywords:** Alzheimer's disease, tau, phosphorylation, iron deposition, insulin resistance

## Abstract

Iron deposition in the brain is an early issue in Alzheimer's disease (AD). However, the pathogenesis of iron-induced pathological changes in AD remains elusive. Insulin resistance in brains is an essential feature of AD. Previous studies determined that insulin resistance is involved in the development of pathologies in AD. Tau pathology is one of most important hallmarks in AD and is associated with the impairment of cognition and clinical grades of the disease. In the present study, we observed that ferrous (Fe^2+^) chloride led to aberrant phosphorylation of tau, and decreased tyrosine phosphorylation levels of insulin receptor β (IRβ), insulin signal substrate 1 (IRS-1) and phosphoinositide 3-kinase p85α (PI3K p85α), in primary cultured neurons. In the *in vivo* studies using mice with supplemented dietary iron, learning and memory was impaired. As well, hyperphosphorylation of tau and disrupted insulin signaling in the brain was induced in iron-overloaded mice. Furthermore, in our *in vitro* work we identified the activation of insulin signaling following exogenous supplementation of insulin. This was further attenuated by iron-induced hyperphosphorylation of tau in primary neurons. Together, these data suggest that dysfunctional insulin signaling participates in iron-induced abnormal phosphorylation of tau in AD. Our study highlights the promising role of insulin signaling in pathological lesions induced by iron overloading.

## Introduction

Alzheimer's disease (AD) is a devastating brain disorder and is the most common cause of dementia in the elderly. AD is characterized clinically by cognitive impairment, and pathologically by amyloid beta (Aβ) deposition as well as aberrant phosphorylation of tau in the brain (1). Familial AD, also known as the early onset AD, affects <5% of all AD individuals and is modulated by genetic mutations (2). However, the majority (i.e., 95%) of AD cases are due to sporadic or late onset AD (2).

Neurofibrillary tangles (NFTs) comprise paired helical filaments of hyperphosphorylated tau. In neurons, NFTs lead to cytoskeletal disruption that subsequently results in neural damage and loss of function (3). Previous studies hypothesized that in the amyloid cascade, Aβ was the igniter, leading to pathological changes including hyperphosphorylation of tau in sporadic AD (SAD) (4). However, the role of Aβ remains controversial since other evidence suggests that tau is a necessary factor in Aβ-induced neurotoxicity (5). Indeed, tauopathy is well-correlated with cognitive impairment and clinical grades of AD, but without significant senile plaque (SP) deposition (6, 7), suggesting the importance of tauopathy in the development of AD. Physiologically, the microtubule-associated protein tau carries out multiple critical roles in the neuron, such as morphogenesis, polarity, plasticity, and cytoskeleton stabilization (8, 9). Despite extensive research, the precise etiopathogenesis and pathophysiologic mechanism of SAD is still unclear and disease-modifying therapeutic management is not currently available (10).

Metals such as iron, copper, and zinc are essential elements that are indispensable for all normal cellular activity (11). Accumulating evidence indicates that brain homeostasis of transition metals is significantly disrupted in AD, and in particular, iron dyshomeostasis is an early factor the disease progression (12–15). Iron is a critical factor that participates in the physiological function of neurons (16). Investigations based on magnetic resonance imaging (MRI) and anatomical analysis reveal elevated iron deposition in the brains of AD patients (17, 18). The excessive accumulation of iron in the hippocampus is positively correlated with the deposition of SP, and is negatively associated with memory loss in AD (19–21). Importantly, the iron chelating agent deferomine (DFO) alleviates the pathological lesions including Aβ accumulation, hyperphosphorylation of tau, and behavioral disruption, in an iron-overloaded AD model (22, 23), which indicates the essential role of iron in the pathogenesis of AD progression.

In peripheral tissue, the insulin signaling pathway also plays a pivotal role in the modulation of energy homeostasis, and in maintaining the normal neurological function in the central nervous system (CNS) (24–26). Insulin receptor and its target molecules are wildly distributed and this receptor acts as a crucial neuromodulator in the brain (26). Because of the association between AD and diabetes, studies were undertaken on the effect of dysfunctional insulin signaling in AD progress, which led to subsequent studies that showed brain insulin resistance is a hallmark of AD (27–29). Interestingly, it was previously reported that in AD patients, insulin receptor sensitivity is reduced, and the levels of phosphorylation of the insulin receptor, and its substrates, are decreased in the brain. However, the content of insulin in the cerebrospinal fluid (CSF) does not change, or is mildly elevated in age-matched healthy individuals (30–32). Furthermore, it was demonstrated that the treatment with intranasal insulin improves learning and memory in individuals with mild cognitive impairment (MCI) and AD, suggesting the involvement of dysfunctional insulin signaling as a key player in the pathogenic mechanism of AD progression.

The relationship between insulin signaling and tau phosphorylation has been reported in previous studies, which suggested a biphasic role of insulin signaling in the aberrant phosphorylation of tau (33–35). Treatment of insulin within a short time led to a rapid and transient hyperphosphorylation of tau (33, 34). Contrastly, prolonged exposure to insulin resulted in a significant decrease in tau phosphorylation (34, 35). Furthermore, investigations also reveal that insulin resistance in neurons promotes the phosphorylation level of tau (36–38), and in turn hyperphosphorylation of tau also causes dysfunctional insulin signaling (39, 40). However, whether insulin resistance is linked with iron overloading and the pathological changes in AD, remains to be further investigated. Therefore, in this current study, we investigated the effect of iron on the phosphorylation of tau, and on insulin in neurons. We also analyzed these lesions in the brains of mice treated with high-iron chow. Using an *in vitro* model, we then investigated the effect of iron-overloaded neurons following exogenous supplementation of insulin to evaluate the potential role of insulin signaling in iron-induced aberrant phosphorylation of tau in AD progression.

## Materials and Methods

### Cell Culture and Treatments

Primary neurons were isolated, purified, and then cultured as previously described, with some modifications to the methodologies described previously (41, 42). Briefly, on embryonic day 17, pregnant Sprague-Dawley (SD) rats were anesthetized and the fetuses collected to isolate cortices for digestion using trypsin (Gibco, USA). Cell suspensions were filtered, centrifuged, and then plated onto poly-L-lysine-coated dishes or plates. Cells were cultured in a 60 mm dish for protein determination using Western blot, in 24-well plates for immunofluorescence staining, and in 96-well plates for the cell viability assay. After 4 h, the medium was replaced with Neurobasal medium (Gibco, USA) containing B27 and GlutaMAX (Gibco, USA). The cultures were maintained at 37°C in a humidified 5% CO_2_ atmosphere for 12 days before treatment.

Ferrous (Fe^2+^) chloride (Sigma, USA) was used to achieve iron overloading in cultures. Ferrous (Fe^2+^) chloride powder was dissolved in the solvent of sterilized water containing 0.01 N HCl as previously described, and a final concentration of 20 μM in the cultures was used, as we reported elsewhere (42), where an isometric solvent was delivered into the cultures as a normal control.

For treatment with insulin, bovine insulin (Sigma, USA) was solubilized at 1 mg/ml as a stock solution and frozen at −20°C in single-use aliquots. The final concentration of insulin was 1 μg/ml as previously reported (43) and incubation was for 24 h, after which the cells were analyzed.

### Cell Viability Detection

To determine cell viability, the CKK-8 assay kit (Dojindo, Japan) was used and the procedures were conducted according to the manufacturer's instructions. A microplate reader set at a wavelength of 450 nm (Thermo Fisher, USA) was used to measure the absorbance.

### Animals and Treatments

Animal procedures were approved by the Medical Experimental Animal Administrative Committee of Renji Hospital, Shanghai Jiao Tong University School of Medicine, Shanghai, China.

Two-month-old male C57BL/6 mice were purchased from Shanghai SLAC (China). Mice were housed with unrestricted access to food and water in a 22°C environment that maintained a 12–12 h light-dark cycle. For the iron overloading model, mice were fed with high-iron chow (3,000 mg carbonyl iron/kg diet, TROPHIC, China) as previously reported (44, 45). Control mice were fed standard chow (50 mg iron/kg diet, TROPHIC, China). Six weeks later, the mice were sacrificed for experimental investigation.

### Morris Water Maze Test

The Morris water maze (MWM) was conducted as previously reported (46–48). Initially, mice (*n* = 8/group for each independent experiment) were trained twice each day to find the concealed platform in the maze. The trial was ended if the mice successfully climbed onto the escape platform, or after 60 s. Each mouse could remain on the platform for 15 s. The training persisted for 5 days and the platform was removed on the sixth day. In the probe test, mice were tracked, and parameters were recorded including escape latency, cross time, target quadrant, and percentage of time in target quadrant.

### Brain Tissue Preparation

Mice were deeply anesthetized by inhaling isoflurane and transcardially perfused with normal saline. For Western analysis and iron level detection, mice (*n* = 3/group for each independent experiment) were then decapitated, and the cerebral cortex of each mouse was quickly collected and frozen for further analysis. For immunofluorescence staining, mice (*n* = 3/group for each independent experiment) were then fixed with a freshly prepared solution of 4% paraformaldehyde (PFA; Sigma, USA) in 0.1 M PB (pH 7.2). After decapitation, the skull was removed and the whole brain was successively immersed in PFA and then sucrose, successively each for 24 h. Serial 20 μm thick coronal tissue sections were obtained using a freezing microtome (Leica, USA).

### Detection of Iron in the Brain

Iron levels in mouse brains were detected using an Enzyme Linked Immunosorbent Assay kit from Abcam Company as previously reported (49). The process was conducted according to the manufacturer's protocol.

### Co-immunoprecipitation

IRβ and IRS-1 protein interaction was determined using co-immunoprecipitation (Co-IP) as previously described (50). Briefly, lysates from neural cultures were incubated with the primary antibodies overnight on a rotator at 4°C, followed by addition of Protein A and G Agarose (Roche, CH) to capture the immune-complexes. Then, the agarose-antibody-complexes were eluted and centrifuged for collection. Subsequently, the complexes were mixed with 50 μL of 2 × SDS loading buffer. Finally, Western blot analysis was performed to detect the presence of targeted proteins.

### Western Blot

The procedure of Western blotting (51, 52) was carried out as we described previously (48, 53). Briefly, the protein concentration was determined using the Pierce BCA Protein Assay Kit (Thermo fisher, USA) and protein samples (30 μg total protein per lane) were loaded onto a 10% sodium dodecyl sulfate–polyacrylamide gel and electrophoresed (SDS-PAGE). Then, fractionated proteins were transferred onto nitrocellulose (NC) membrane, followed by incubation overnight at 4°C with the indicated antibodies (Table 1). Following washing of the membranes, they were incubated with a secondary antibody (IRDye®680LT, IRDye®800CW, 1:10,000 dilution, LI-COR) for 1 h at room temperature. Images were captured, and band intensities measured using the Odyssey infrared fluorescence imaging system (LI-COR, USA).

**Table 1:** Primary antibodies employed in this study.

**Antibody**	**Type**	**Dilution**	**Source**	**Cat. No**.
IRβ	Rabbit monoclonal	1:1,000	CST	3025
pIRβ Y1150/1151	Rabbit monoclonal	1:1,000	CST	3024
IRS-1	Rabbit polyclonal	1:1,000	CST	2382
pIRS-1 Y612	Rabbit polyclonal	1:1,000	CST	2386
PI3K p85α	Rabbit monoclonal	1:1,000	CST	4257
pPI3K p85α Y458	Rabbit polyclonal	1:1,000	CST	4228
pTau T181	Rabbit polyclonal	1:1,000	CST	5383
pTau S396	Mouse monoclonal	1:1,000	CST	9632
AT8 (S202/T205)	Mouse monoclonal	1:1,000	Pierce	MN1020
Tau5	Mouse monoclonal	1:1,000	Abcam	ab80579
GAPDH	Rabbit polyclonal	1:2,000	Santa	sc-25778

### Immunofluorescence Staining

For brain tissue, the sections were washed with PBS, and this was followed by incubation with 10% goat serum in PBS containing 0.5% Triton-X 100 for 1 h at 37°C. Subsequently, the sections were incubated with AT8 antibody (mouse monoclonal, 1:1,000, Pierce, USA) for 1 h at 37°C followed by overnight at 4°C. After incubation for 1 h at 37°C with the secondary antibodies (Alexa goat anti-rabbit 555 and Alexa goat anti-mouse 555, Invitrogen, USA), the sections were stained with DAPI (1:10,000, Invitrogen, USA) to detect nuclei. Images were acquired with a fluorescence microscope (Nikon, Japan). Fluorescence intensity was measured using Image-Pro Plus, Version 6.0 (MediaCybernetics, Inc., USA) and was normalized to the number of neurons.

For cells maintained on a slide, the slide was washed with 0.01 M PBS and then the cells were fixed with 4% PFA as we described elsewhere (54). After washing with PBS, the cells were then incubated with 10% goat serum in PBS containing 0.5% Triton-X 100 for 1 h at 37°C. Then the subsequent procedures were as described for brain sections above and the fluorescence intensity was normalized to the brain surface.

### Statistical Analyses

Results are shown as the mean ± SD. Statistical analysis was carried out using GraphPad Prism 5 (GraphPad Software, Inc. USA). Experiments were repeated on three independent occasions. In the test of MWM, the number of mice was eight in each group for each independent experiment. In the biochemical detection and fluorescence staining, the number of mice was three in group for each independent experiment. The statistical significance of differences among the various groups was examined using one-way analysis of variance (ANOVA) or the student *t*-test. A value of *p* < 0.05 was considered to be statistically significant.

## Results

### Elevated Levels of Phosphorylated Tau in Iron-Treated Neurons

**Figure 1:**
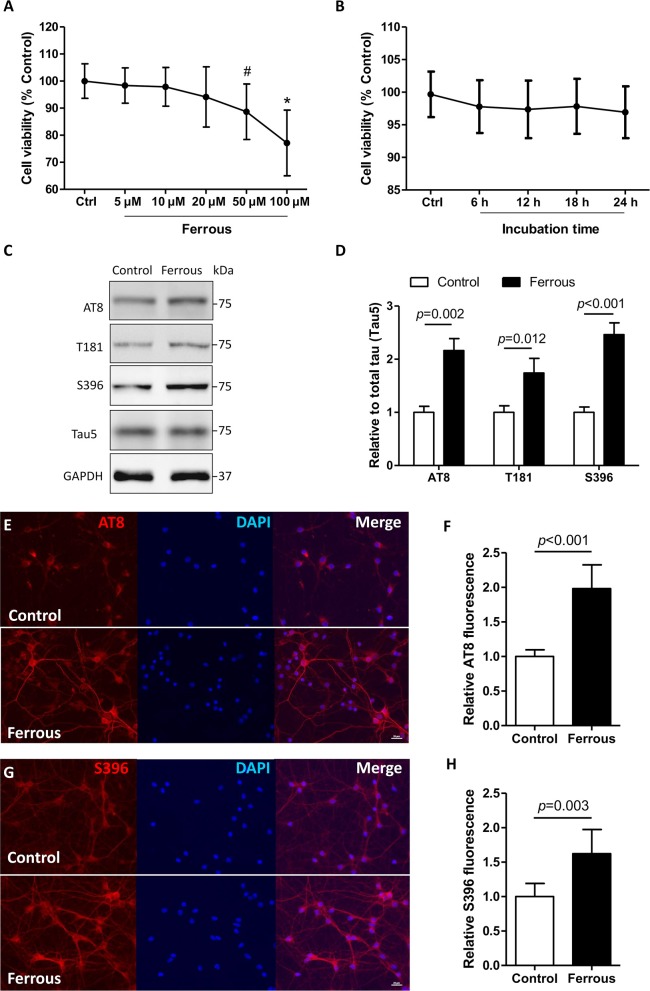
Phosphorylation levels of tau are increased in iron-treated neurons. In primary cultures, neurons were treated with ferrous (Fe^2+^) chloride for 24 h and then analyzed by various of assays. **(A)** Shows the cell viability data after treatment with different concentrations of (Fe^2+^) chloride for 24 h. **(B)** Shows the cell viability result when neurons were treated 20 μM ferrous (Fe^2+^) chloride at different time points. **(C)** The levels of tau protein phosphorylation at epitopes of AT8, T181, and S396 were determined using Western blots. **(E,G)** Show immunofluorescence staining revealing an increase in the level of phosphorylation at epitopes AT8 and S396 in neurons, respectively. **(D,F**,**H)** Show the quantitation of the results presented in **(C**,**E**,**G)**, respectively. Results are shown as mean ± SD. Student's *t*-test was employed to determine the significance. *NS*, no significant difference. ^*^vs. Control, *p* < 0.01, ^#^vs. Control, *p* < 0.05.

Iron deposition results in a decrease in tau phosphorylation (55). However, the phosphorylation level of tau was increased in the brains of dietary iron-treated mouse (22) and in neurons exposed to ferrous (Fe^2+^) chloride (56). In neurons, iron is delivered across the neural membrane primarily by transferrin–transferrin receptor system-mediated uptake and efflux, via ferroportin (Fpn) in the form of ferrous (Fe^2+^) chloride (14). Therefore, in our *in vitro* work neurons were treated with ferrous (Fe^2+^) chloride, and in the *in vivo* study the mice were administered a diet containing carbonyl iron (Fe^0^). As previously described (42), ferrous (Fe^2+^) chloride was employed to model the microenvironment of iron deposition. After a 24 h treatment with either 50 μM or 100 μM ferrous (Fe^2+^) chloride, the viability of neurons was significantly reduced by 23 and 11%, respectively (Figure 1A, *p* < 0.05). At a concentration of 20 μM, the ferrous (Fe^2+^) chloride treatment led to a decrease of 5% in cell viability after treatment for 24 h in primary cultured neurons, but this decrease was not statistically significant (Figures 1A,B).

In AD, multiple characteristic epitopes of tau protein phosphorylation have been reported including Ser202/Thr205 (AT8), Thr181 (T181), and Ser396 (S396) (57, 58). In iron-treated neurons, the levels of tau phosphorylation at epitopes of AT8, T181, and S396 were elevated by 1.2-, 0.7-, and 1.5-fold, respectively (Figures 1C,D, *p* < 0.05, *p* < 0.01). Using immunofluorescence staining, we also observed increased levels of phosphorylation at epitopes of AT8 and S396 in ferrous (Fe^2+^) chloride-incubated neurons (Figures 1E–H, *p* < 0.01).

### Treatment With Iron Disrupts Insulin Signaling in Neurons

Previous studies suggested an essential role of insulin resistance in the hyperphosphorylation of tau, but whether insulin resistance contributes to iron-induced tauopathy has not been determined. Here, we evaluated the changes in insulin signaling in iron-treated neurons. In physiological conditions, extracellular insulin combines with, and activates, insulin receptor α (IRα). This successively leads to tyrosine phosphorylation of both insulin receptor β (IRβ) and insulin signaling substrates (IRS), which activates and engages the insulin signaling pathway (59).

**Figure 2:**
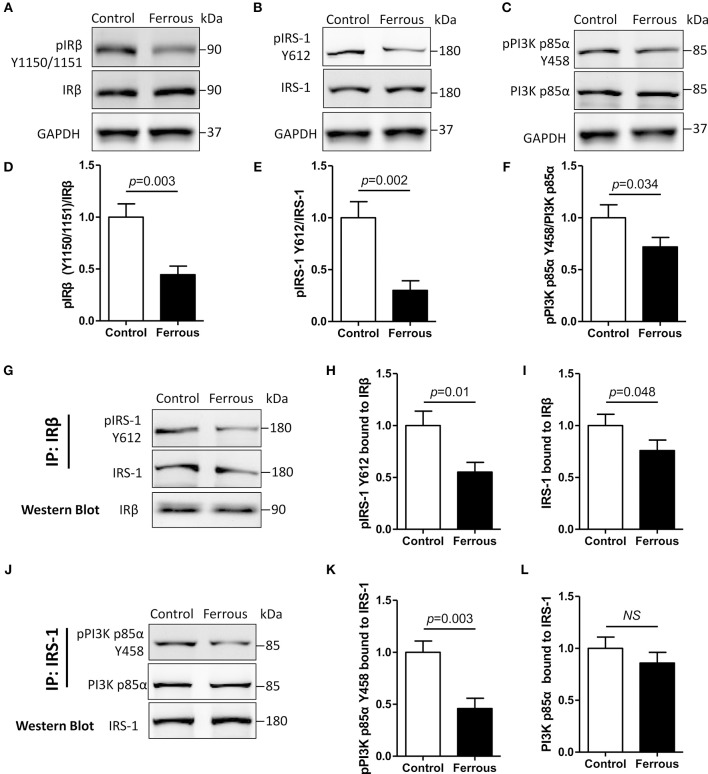
Dysfunctional insulin signaling is induced by the treatment of neurons with iron. Changes of in tyrosine phosphorylation, and total IRβ, IRS-1, and PI3K p85α protein, were evaluated using Western blot **(A–C)**. Antibodies recognizing IRβ **(G)** or IRS-1 **(J)** were utilized for co-immunoprecipitation with total proteins, and then the predicated proteins were determined using Western blot. **(D–F)** Show the quantitation of the results presented in **(A–C)**, respectively. **(H**,**I)** Show the quantitation of the results presented in **(G)**. **(K,L)** Show the quantitation of the results presented in **(J)**. Results are shown as Mean ± SD. Student's *t*-test was employed to determine the significance. *NS*, no significant difference.

We were unable to detect any changes in the level of unphosphorylated IRβ (i.e., total IRβ protein content) in iron-treated neurons, but the tyrosine phosphorylation content of the IRβ kinase regulatory domain (pIRβ Y1150/1151) was significantly decreased by 55% as compared to the control (Figures 2A,D, *p* < 0.01). With regard to downstream factors of insulin signaling, the tyrosine phosphorylation levels of both IRS-1 (pIRS-1 Y612) and phosphoinositide 3-kinase p85α (pPI3K p85α Y458) were also significantly decreased by 70 and 28%, respectively (Figures 2B,C,E,F, *p* < 0.01), but the total protein content of either of the two targets was not changed after 24 h incubation with iron (Figures 2A–C).

We then evaluated protein-protein interactions in insulin signaling using the co-immunoprecipitation (Co-IP) assay (50). IRβ-bound total protein, and tyrosine phosphorylation levels of IRS-1, were decreased by 45 and 24%, respectively, in iron-treated neurons (Figures 2G–I, *p* < 0.05, *p* < 0.01). Treatment with iron caused a reduction in the level of IRS-1-bound total PI3K p85α, which was not significant (Figures 2J,L), but also led to a significant 54% decrease in IRS-1-bound tyrosine phosphorylation of PI3K p85α (Figures 2J,K, *p* < 0.01).

### Supplemental Dietary Iron Impairs Cognition and Causes Abnormal Phosphorylation of Tau in Mice

**Figure 3:**
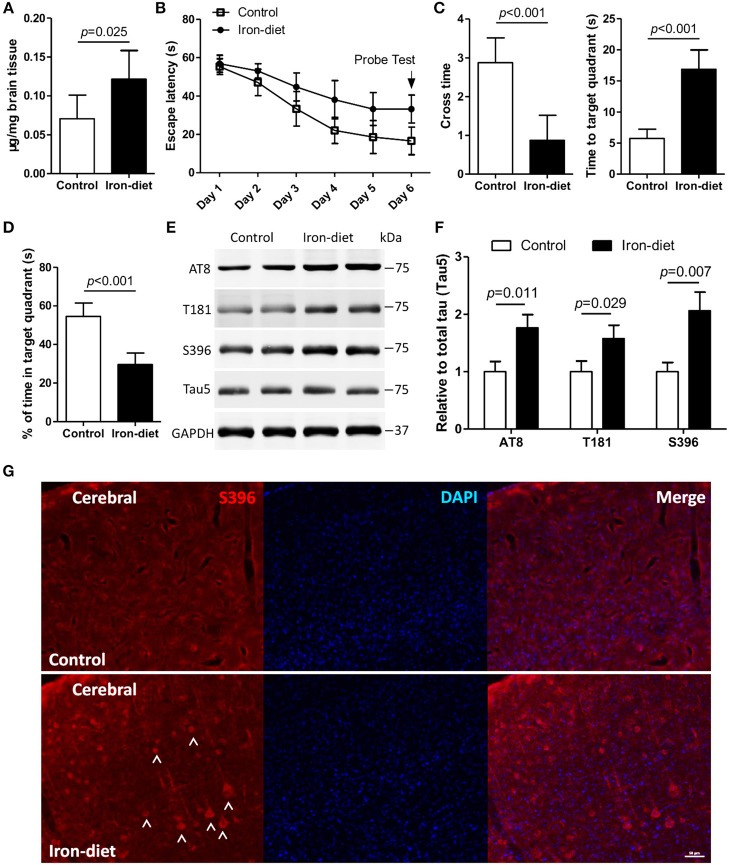
Cognition is impaired, and there is aberrant phosphorylation of tau in iron-overloaded mouse brains. In the MWM test, mice were trained for 5 days and then a probe test (i.e., the platform was removed) was performed on the sixth day. The content of iron in the mouse brains was determined **(A)**. The escape latency **(B)**, cross time and time to target quadrant **(C)**, and the percentage of time in target quadrant **(D)** were evaluated. The levels of tau phosphorylation in iron-overloaded mouse brains was determined using Western blot analysis **(E)**, and immunofluorescence staining **(G)**. In the MWM test, the number of mice was eight in each group for each independent experiment. In biochemical detection and fluorescence staining, the number of mice was three in group for each independent experiment. **(F)** shows the quantitation of the results presented in **(E)**. Results are shown as mean ± SD. Student's *t*-test was employed to determine the significance.

To achieve iron overloading in the brain, mice were administered a high-iron diet as previously reported (44, 45). As shown in Figure 3A, the contents of mouse brains were increased by 0.71-fold after treatment with a high-iron diet for 6 weeks (*p* < 0.05). We evaluated the learning and memory function in iron-overloaded mice. In the MWM test, the escape latency in iron-overloaded mice was increased by 2.7-fold, and the cross time was significantly decreased as compared to the control (Figures 3B,C, *p* < 0.01). Additionally, the data representing the time to the target quadrant, and the percentage of time in the target quadrant, showed impaired learning and memory in iron-overloaded mice (Figures 3C,D, *p* < 0.01).

We subsequently investigated whether the pathology associated with aberrant tau phosphorylation was induced in mice fed with high-iron chow. In iron-treated mice, the content of total tau, determined with Tau5 antibody using Western blot, was not changed. However, the levels of tau phosphorylation at multiple epitopes, including AT8, T181, and S396, were increased by 0.76-, 0.57-, and 1.1-fold, respectively, in the brains of iron-overloaded mice (Figures 3E,F, *p* < 0.05, *p* < 0.01). These results revealed that alteration of epitopes AT8 and S396 was significant after treatment with a high-iron diet (Figures 3E,F, *p* < 0.05, *p* < 0.01). An independent, immunofluorescence staining assay provided evidence of an increase in the level of phosphorylation at epitope S396 in iron-overloaded mice brains (Figure 3G), which is consistent with increased S396 phosphorylation detected in iron-treated neurons (Figures 1C–H).

### Insulin Signaling Is Disrupted in the Brains of Mice Fed With High-Iron Chow

**Figure 4:**
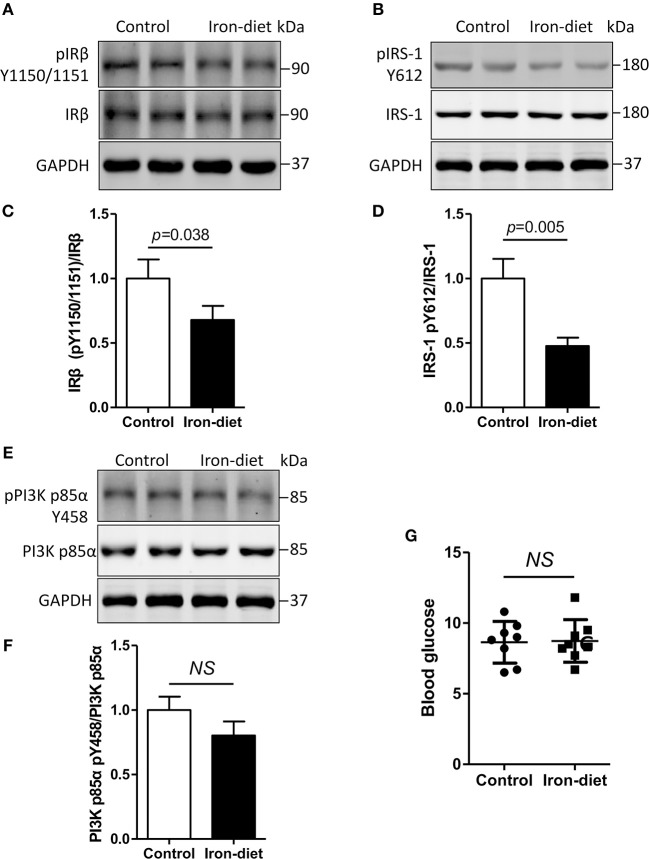
Insulin signaling is disturbed in iron-overloaded mouse brains. The changes of insulin signaling in mouse cerebral cortexes were investigated using Western blot analysis. The total protein content of IRβ and IRS-1 was not altered. However, the tyrosine phosphorylation levels of IRβ and IRS-1 were decreased in iron-overloaded mouse brains **(A,B,E)**. Total PI3K p85α protein in iron-overloaded mouse brains was not changed. A decrease in tyrosine phosphorylated PI3K p85α Y458 was observed but was not significant **(E)**. In the analysis, the number of mice was three in group for each independent experiment. **(C,D,F)** Show the quantitation of the results presented in **(A**,**B**,**E)**, respectively. **(G)** Shows the blood glucose levels that were determined using a glucometer. Results are shown as mean ± SD. Student's *t*-test was employed to determine the significance.

As previously mentioned, insulin signaling was disrupted by the treatment of iron *in vitro*. We then evaluated the phosphorylation changes of IRβ, IRS-1, and PI3K p85α *in vivo*. As shown in Figure 4, the levels of unphosphorylated IRβ, IRS-1, and PI3K p85α were equivalent in iron-overloaded mouse brains compared with controls. Tyrosine phosphorylation levels of IRβ (pIRβ Y1150/1151) and IRS-1 (pIRS-1 Y612) were decreased by 32 and 52%, respectively (Figures 4A–D, *p* < 0.05, *p* < 0.01). We also determined the levels of PI3K p85α in iron-overloaded mouse brains and found that the total level of PI3K p85α was not altered (Figure 4E). However, there was a 15% reduction of tyrosine phosphorylated PI3K p85α (pPI3K p85α Y458), but this was not statistically significant (Figures 4E,F). Furthermore, we also determined the change of blood glucose in iron-overloaded mice and the result showed that there was also no significant difference between the levels of blood glucose in the two groups (Figure 4G).

### Abnormal Phosphorylation of Tau Due to Iron Overloading Is Attenuated in Insulin-Treated Neurons

**Figure 5:**
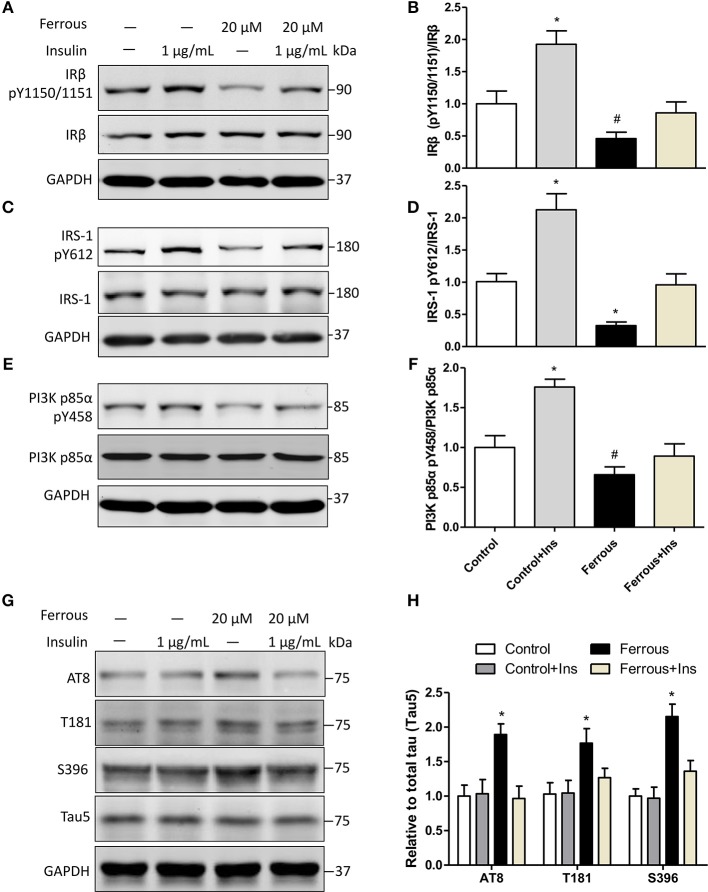
Activation of insulin signaling attenuates the effect of iron-induced hyperphosphorylation tau in primary neurons. Exogenous 1 μg/ml insulin was added to neurons, together with exposure to iron. Twenty-four hours later, neurons were collected and analyzed by Western blot. **(A**,**C**,**E)** show the changes in insulin signaling in neurons, consistent with activated insulin signaling. Phosphorylation levels of tau were determined **(G)**. **(B,D**,**F**,**H)** show the quantitation of the results presented in **(A**,**C**,**E**,**G)**, respectively. Results are shown as mean ± SD. One-way ANOVA was employed to determine the significance. ^*^vs. Ctrl, *p* < 0.01, ^#^vs. Ctrl, *p* < 0.05.

To further investigate the role of dysfunctional insulin signaling resulting from iron-induced changes to tau protein, neurons were incubated with supplementary insulin, with or without the treatment of ferrous (Fe^2+^) chloride. The effect of supplementary insulin on insulin signaling was analyzed by determining the tyrosine phosphorylation levels of targets. Western blot analysis showed that supplementary insulin did not alter the total protein levels of IRβ, IRS-1, andPI3K p85α (Figures 5A,C,E), but supplementary insulin raised the phosphorylation levels of pIRβ Y1150/1151, pIRS-1 Y612, and pPI3K p85α Y458 in neurons incubated with or without ferrous (Fe^2+^) chloride (Figures 5A–E). However, the increase in tyrosine phosphorylation levels in iron-treated neurons remained reduced compared to the controls (Figures 5A–F).

Then, we determined the changes of taupathy after exposure to insulin. We found that insulin signaling was activated by exogenous addition of insulin, but the levels of phosphorylated tau were not changed in neurons without iron treatment (Figures 5G,H). This suggested that 1 μg/ml insulin was a suitable concentration in our work and the insulin resistance was not induced in neurons. In iron-treated neurons, increased phosphorylation at AT8, T181, and S396 was ameliorated by supplementation with insulin (Figures 5G,H).

## Discussion

Almost all organisms and cells require iron to act as a cofactor in cellular processes such as energy metabolism, DNA synthesis, and repair, oxygen transport, signal transduction, electron transport, and neurotransmitter transmission (16, 60). The constant balance of uptake and elimination is critical for iron homeostasis not only at the whole human body level, but also in each organ and cell (61). Disruption of iron metabolism is implicated in the etiology of many diseases including neurodegenerative disorders. In the brain, iron in the blood circulation is captured mainly via the transferrin- transferrin receptor 1 (TFR1) system, and then crosses the endothelial cells of blood-brain barrier (BBB), to then be taken up by other cells such as neurons and astrocytes (62). In neurons, the influx of iron is mediated mostly by TFR, and efflux via ferroportin (Fpn) (62), but the detailed mechanism is not fully known.

In the past few decades, studies have provided increasing support for a correlation between iron deposition in the brain and neurodegenerative diseases such as AD (63–65). By MRI detection, the level of iron in the brain correlates with cognitive impairment (66). The current study investigated the potential role of intracerebral iron-overloading in taupathology and alteration of insulin signaling. In this study, we first evaluated cognitive function and observed that iron overloading impaired learning and memory in mice. However, patients with AD do not generally adhere to iron-rich diets and iron levels in the blood are normal (67), which suggests that perturbed iron distribution is due to a neuronal imbalance between the influx and efflux of iron.

In AD, iron overloading in the brain is a key pathological feature, together with hyperphosphorylation of tau (20), but the underlying mechanism is not yet fully understood. Previous studies show oxidative stress is involved in iron-induced pathologies (68). Oxidative stress is caused by an imbalance between the generation of reactive oxygen species (ROS) and the detoxifying system in cell (69). Elevated iron levels in neurons lead to the generation of ROS and cause oxidative stress injury (70). Nevertheless, there is controversy regarding the role of oxidative stress, and recent investigations provide evidence that oxidative stress might not be the critical process in iron-induced pathological damage (42, 71). Thus, other mechanisms might also be involved.

Perturbed brain metabolism is an early and invariant characteristic of AD (72, 73). Mounting evidence suggests that cerebral hypometabolism is involved in the development of AD and contributes to the formation of its hallmarks (74–76). Determining the key factors and pathways that lead to dysfunctional cerebral metabolism is essential to determining the mechanisms of AD and for identifying possible targets for intervention. Insulin signaling is the prominent modulator of cellular activities through its regulation of energy metabolism, mitogenesis, development, synaptic plasticity, and other processes (24, 28). Accumulating evidence reveals that dysfunctional insulin signaling is a major risk factor for the onset of AD, particularly the sporadic type (77). Currently, there is no direct evidence of a role for iron in neural insulin signaling. In our study, we observed that tyrosine phosphorylation of particular proteins involved in insulin signaling was decreased in ferrous (Fe^2+^) chloride-exposed neurons, and in iron-overloaded mouse brains. Moreover, aberrant phosphorylation of tau in neurons as a result of exposure to ferrous (Fe^2+^) chloride was reduced by supplementary insulin.

In patients, biomarkers of insulin resistance in the CNS are universally and progressively increased from MCI to AD (50). Furthermore, the severity of insulin resistance is also negatively correlated with cognition, and positively associated with amyloid deposition (50). Insulin resistance markedly impacts on the generation of Aβ and the status of phosphorylation in rodent models of AD (78–81). Targeting the improvement of insulin signaling may ameliorate learning and memory, and inhibit Aβ plaque and NFT formation (38, 82, 83). In patients with amnestic MCI or AD, delivery of insulin intranasally improved delayed memory and functional ability (84).

However, previous work revealed that Ca^2+^ signaling was also modulated by the treatment of iron, which in turn leading to the activation of MAPK/ERK pathway (85). Both Ca^2+^ signaling and MAPK/ERK pathway have been revealed to involved in the progress of AD (86, 87), but their accurate effect in iron-induced tau pathology is still unclear. Thus, further investigations needed to better understand the role of iron deposition in AD.

The present work revealed that dysfunctional insulin signal is involved in the hyperphosphorylation of tau, as a result of iron-overloading. However, we did not further evaluate the effect of aberrant phosphorylation of tau on insulin signal. The relationship of tau pathology and insulin signal is complicated and more information could be found in Gratuze's review which considered the mutual relationship between hyperphosphorylation tau and dysfunctional insulin signal in AD (88). These findings suggest a potential role for insulin signaling in pathological lesions induced by iron accumulation. However, there are limitations in our current work and further investigations are needed. For example, the role of dysfunctional insulin signaling in the *in vivo* tau pathology is unclear and needs further elucidation. Additionally, the effect of iron chelators should be evaluated in iron-overloaded mice, and the mechanisms underlying iron-induced aberrant phosphorylation of tau also need to be determined.

## Ethics Statement

Animal procedures were approved by the Medical Experimental Animal Administrative Committee of Renji Hospital, Shanghai Jiao Tong University School of Medicine, Shanghai, China.

## Author Contributions

WW and LC carried out the experiment and wrote the manuscript with support from YG and BK. YG helped supervise the project. BK, PM, and SX participated in the English language correction.

### Conflict of Interest Statement

The authors declare that the research was conducted in the absence of any commercial or financial relationships that could be construed as a potential conflict of interest.
